# Reduced frequency of migraine attacks following coronavirus disease 2019: a case report

**DOI:** 10.1186/s13256-023-03795-3

**Published:** 2023-02-22

**Authors:** Roland Houben

**Affiliations:** Department of Dermatology, Venereology Und Allergology, University Hospital Würzburg, Josef-Schneider-Str. 2, 97080 Würzburg, Germany

**Keywords:** Migraine, Triptan, Severe acute respiratory syndrome coronavirus 2, Coronavirus disease 2019, Case report

## Abstract

**Background:**

Severe acute respiratory syndrome coronavirus 2 is a virus affecting different organs and causing a wide variety and severity of symptoms. Headache as well as loss of smell and taste are the most frequently reported neurological manifestations of coronavirus disease 2019 induced by severe acute respiratory syndrome coronavirus 2. Here we report on a patient with chronic migraine and medication overuse headache, who experienced remarkable mitigation of migraine following coronavirus disease 2019.

**Case presentation:**

For many years prior to the severe acute respiratory syndrome coronavirus 2 infection, a 57-year-old Caucasian male suffered from very frequent migraine attacks and for control of headaches he had been taking triptans almost daily. In the 16-month period before the outbreak of coronavirus disease 2019, triptan was taken 98% of the days with only a 21-day prednisolone-supported triptan holiday, which, however, had no longer-lasting consequences on migraine frequency. Upon severe acute respiratory syndrome coronavirus 2 infection, the patient developed only mild symptoms including fever, fatigue, and headache. Directly following recovery from coronavirus disease 2019, the patient surprisingly experienced a period with largely reduced frequency and severity of migraine attacks. Indeed, during 80 days following coronavirus disease 2019, migraine as well as triptan usage were restricted to only 25% of the days, no longer fulfilling criteria of a chronic migraine and medication overuse headache.

**Conclusion:**

Severe acute respiratory syndrome coronavirus 2 infection might be capable of triggering mitigation of migraine.

## Introduction

Migraine is a disabling and highly prevalent primary headache affecting more than one billion people worldwide [1]. Genetics, as well as environmental factors, are known to contribute to this neurological disease [2]. Migraine headache is frequently described as severe, throbbing pain on one side of the head, often accompanied by further symptoms such as nausea, vomiting, and sensitivity to light and sound [3].

Coronavirus disease 2019 (COVID-19) is a life-threatening condition caused by the severe acute respiratory syndrome coronavirus 2 (SARS-CoV-2), and affected patients present with a wide variety of symptoms [4]. In this respect, a multitude of neurological manifestations of COVID-19, including headache, have also been reported [5–7]. In particular, headache with migraine characteristic features have been observed in patients with COVID-19 in both the acute and healing phases [8]. While COVID-19 disease was generally described to be associated with either *de novo* headache, or worsening of migraine symptoms, the COVID-19 lockdown has been reported to be associated with both deterioration and also improvement of migraine symptoms [9, 10]. Here we report on a patient with migraine who experienced a 3-month-lasting prominent mitigation of migraine, directly following SARS-CoV-2 infection.

## Case description

Medical history of the 57-year-old Caucasian male patient revealed that he suffered from migraine with aura for almost 40 years. His mother and one of his four children were also affected by the disease. The slim, athletic patient reported that his individual migraine attacks typically persist between 8 and 16 hours and, when left untreated, the unilateral sharp pain severely affects his ability to participate in daily activities. The patient believed that emotional stress, lack of sleep, missed meals, and changing weather may trigger his migraine attacks. While in the early phase of his disease no effective treatment could be established, the headache became more controllable after the advent of the triptans [11]. The patient orally applied sumatriptan in early years, later zolmitriptan (requiring prescription) for very heavy attacks, and naratriptan (available as over-the-counter drug in Germany) for less severe migraine. However, while migraine attacks occurred approximately every 5 days before triptan therapy, the frequency increased since triptan therapy was initiated, finally resulting in almost daily migraine and, as a consequence, almost daily triptan usage. Since 2003 approximately, the patient fulfilled the criteria of medication overuse headache, that is, (1) headache present on more than 15 days/month, (2) regular overuse of a symptomatic treatment (> 10 days per month for triptans) for more than 3 months, and (3) markedly worsened headache during medication overuse [12, 13]. Throughout his adult life, the patient was treated with several prophylactic therapies including beta-blockers, tricyclic antidepressants, topiramate, acupuncture, neural therapy, and psychotherapy, as well as dietary changes and relaxation techniques. Out of these measures topiramate was most effective, significantly reducing migraine frequency. However, this outcome of topiramate treatment was transient. Indeed, the therapy was started several times but then discontinued after a few months or weeks upon return of daily migraine and daily use of triptans. In combination with topiramate, but also with other prophylactic therapies, the patient had—over a period of 13 years—a total of seven prednisolone-supported drug holidays from triptans. However, long-lasting effects on migraine frequency and reduction of triptan overuse could not be achieved, particularly not in recent years.

**Fig. 1:**
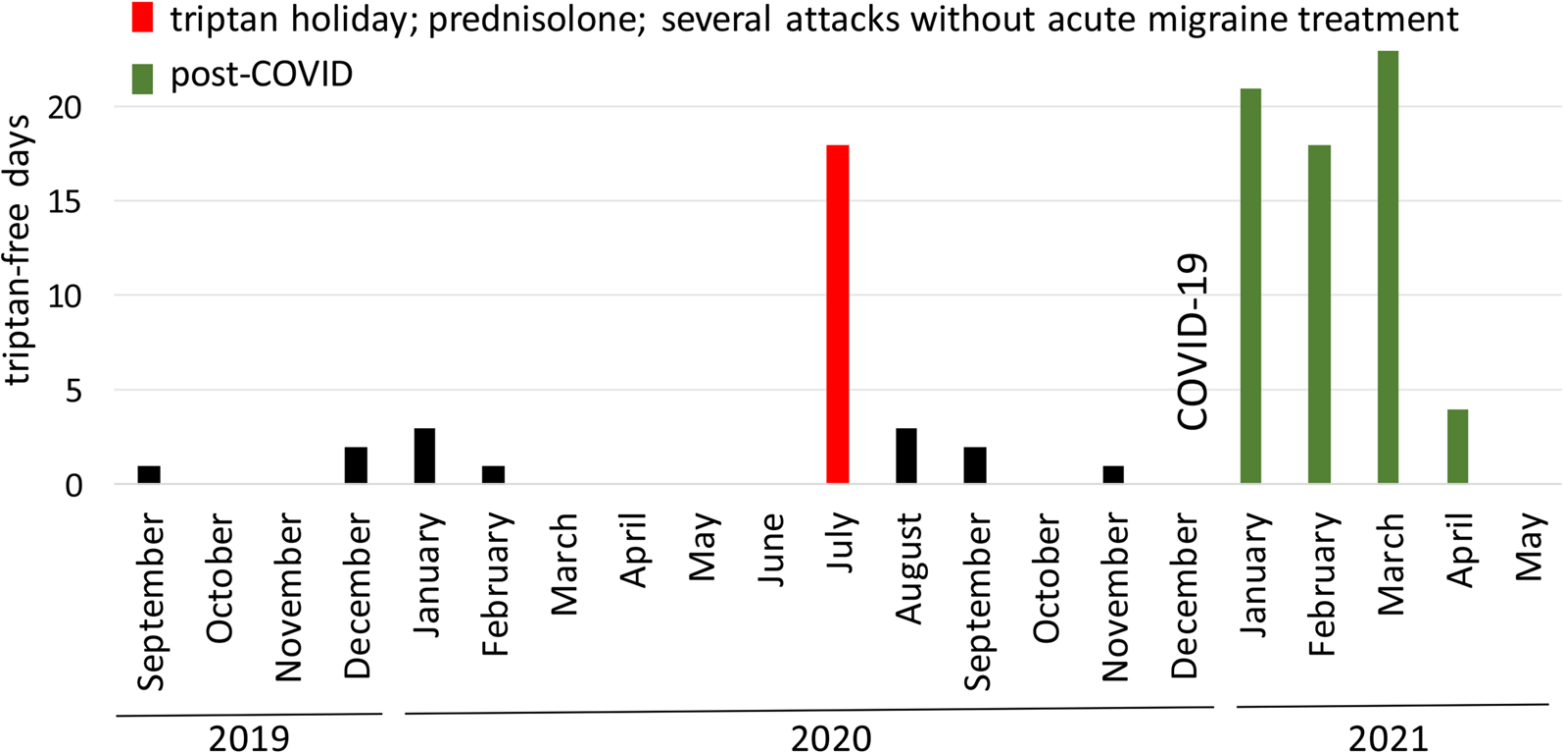
Remarkable increase in triptan-free days directly following COVID-19 disease. Displayed is the number of days per month without triptan consumption from September 2019 to May 2021, based on the recordings of the patient. The number of migraine-free days was identical to the depicted triptan-free days, with the exception of July 2020, when the 57-year-old patient experienced four migraine attacks, without triptan intervention, during a drug holiday associated with 12 days of prednisolone treatment (100 mg on the first 2 days and stepwise reduction to 10 mg on the last two days). The first SARS-CoV-2-positive PCR test was on 21 December 2020. The patient had COVID-19 symptoms until 4 January 2021, and a period of 80 days with only 20 migraine attacks started on 7 January 2021. Starting on 28 March, the patient returned to having almost daily migraine attacks

In December 2020, the patient had extended contact to a SARS-CoV-2-positive person and developed COVID-19 symptoms, and two positive SARS-CoV-2 PCR tests confirmed the infection. The course of his COVID was mild, with fever, fatigue, and headache, as well as weak respiratory symptoms. Headache was bilateral but developed every day into a unilateral migraine attack. However, after approximately 20 days, the patient not only felt as if he had completely recovered from COVID-19, but surprisingly also experienced only three migraine attacks over a period of 24 days. This was unprecedented and, with the exception of periods of high-dose corticosteroid treatment, had not occurred in the previous 15 years—according to the patient’s memory and his written migraine diary covering the last 5 years. Reduced frequency of migraine attacks and triptan usage compared with the pre-COVID-19 phase persisted for 80 days (Fig. 1). During this period, the patient experienced only 20 migraine attacks and 20 days with triptan usage (25% of the days), thus implying that the patient fulfilled the criteria neither for chronic migraine nor for medication overuse headache. Almost as suddenly as the period with reduced migraine had started, it ended with a return to almost daily migraine (Fig. 1).

## Discussion and conclusion

Prevalence of chronic migraine depends on age. The American migraine prevalence and prevention study revealed that for males a prevalence maximum of 0.79% is reached between 40 and 50 years, and that prevalence drops to 0.26% for men above 60 [14]. This may imply that spontaneous regression of the disease is not a very unlikely event for a 57-year-old man and it cannot be excluded that the observed reduced migraine attacks in the reported case are not related to the SARS-CoV-2 infection. Nonetheless, the very strict chronological association of the two events, the rapid and significant reduction of headache episodes, and the transient nature of the phenomenon suggest that the mitigation of chronic migraine was triggered by COVID-19. Given such a relationship, it can, however, not be excluded that the effect on migraine is only secondary to, for example, changes in lifestyle during or after COVID-19 disease. Indeed, it is well known that factors such as sleep, exercise, mealtime pattern, or hydration status may affect migraine occurrence [15]. However, the patient described the COVID-19 disease on the one hand as a stressful period and, on the other hand, with respect to changes in lifestyle, as not significantly different to previous respiratory tract infections, which were not associated with subsequent improvement of migraine symptoms. Moreover, he reported that he could not identify obvious differences in triggers or lifestyle before and after COVID-19. Therefore, a secondary lifestyle-related effect is unlikely to be the cause of migraine mitigation in this case, and it will be interesting to study whether other patients with chronic migraine experienced similar effects on attack frequency following SARS-CoV-2 infection. In this respect, a recent online survey revealed that SARS-CoV-2-infected patients experienced worsening of their headaches amid the infection period, while no information on the post-COVID phase was provided in the report [16]. In contrast, evaluating symptoms of patients with COVID-19 with severe course 7.3 months after hospital discharge, Fernandez-de-Las-Penas *et al.* identified previous history of migraine to be associated with long COVID (for example, fatigue), but not with changes in prevalence of headache [17]. However, since the considered time period following COVID-19, as well as severity of COVID symptoms of the studied group, was quite different, conclusions with respect to the patient reported here can hardly be drawn.

Interestingly, a few cases of COVID-19-related changes in migraine symptoms have been reported. In this respect, usage of surgical face masks was identified as a measure leading to improvement of symptoms of a migraine exclusively triggered by odors [18]. Waliszewska-Prosol *et al.* reported three patients who, after many years of migraine without aura, experienced aura for the first time during their SARS-CoV-2 infection, which in all three cases was associated with anosmia [19]. A link between anosmia and headache is also suggested by a case of persistence of these two symptoms 85 days following start of COVID-19 [20]. To the best of the author’s knowledge, a case of improved migraine symptoms following COVID-19 has not been reported yet.

Attempts to explain how SARS-CoV-2-triggered migraine mitigation might be mediated mechanistically can only be highly speculative, because we have little knowledge on the interaction of SARS-CoV-2 with the nervous system [21]. Moreover, although various complementary hypotheses (for example, activation and increased sensitization of trigeminal nociceptive afferents innervating the cranial meninges and related blood vessels, and meningeal neurogenic inflammation) are well-accepted concepts [22], the molecular pathology of migraine is not completely understood [22, 23]. However, a possible convergence point could be the blood–brain barrier, which has been suggested to be affected during the headache attack of patients with migraine [24]. On the other hand, using brain organoid models, it has been shown that SARS-CoV-2 can damage the choroid plexus epithelium [25]. Therefore, it may be speculated that, during repair of virus-induced blood–brain barrier damage, a migraine-suppressing environment might be produced.

Irrespective of the mechanism, the presented case suggests that SARS-CoV-2 infection might be capable of triggering mitigation of migraine. To evaluate whether this unexpected outcome might be shared with other patients with migraine, it could be worth including questions about possible headache-diminishing consequences of COVID-19 in future survey studies.

## Data Availability

All data generated or analyzed during this study are included in this published article.
